# Synthesis, Stability and Relaxivity of TEEPO-Met: An Organic Radical as a Potential Tumour Targeting Contrast Agent for Magnetic Resonance Imaging

**DOI:** 10.3390/molecules23051034

**Published:** 2018-04-27

**Authors:** Maiju Soikkeli, Kaisa Horkka, Jani O. Moilanen, Marjut Timonen, Jari Kavakka, Sami Heikkinen

**Affiliations:** 1 Department of Chemistry, University of Helsinki, P.O. Box 55, 00014 Helsinki, Finland; maiju-lotta.soikkeli@helsinki.fi (M.S.); kaisa.horkka@ki.se (K.H.); jari.kavakka@storaenso.com (J.K.); 2 Department of Clinical Neuroscience, Karolinska Institutet, S-17176 Stockholm, Sweden; 3 Department of Chemistry, Nanoscience Centre, University of Jyväskylä, P.O. Box 35, 40014 Jyväskylä, Finland; jani.o.moilanen@jyu.fi; 4 HUS Helsinki Medical Imaging Center, Radiology, University of Helsinki and Helsinki University Hospital, P.O. Box 340, 00029 HUS, Helsinki, Finland; marjut.timonen@hus.fi; 5 Stora Enso, Innovation Centre for Biomaterials, Fannys väg 1, S-13154 Nacka, Sweden

**Keywords:** nitroxides, radicals, methionine, contrast agents, MRI, NMR, EPR

## Abstract

Cancer is a widespread and life-threatening disease and its early-stage diagnosis is vital. One of the most effective, non-invasive tools in medical diagnostics is magnetic resonance imaging (MRI) with the aid of contrast agents. Contrast agents that are currently in clinical use contain metals, causing some restrictions in their use. Also, these contrast agents are mainly non-specific without any tissue targeting capabilities. Subsequently, the interest has notably increased in the research of organic, metal-free contrast agents. This study presents a new, stable organic radical, TEEPO-Met, where a radical moiety 2,2,6,6-tetraethylpiperidinoxide (TEEPO) is attached to an amino acid, methionine (Met), as a potentially tumour-targeting moiety. We describe the synthesis, stability assessment with electron paramagnetic resonance (EPR) spectroscopy and relaxation enhancement abilities by an in vitro nuclear magnetic resonance (NMR) and phantom MRI studies of TEEPO-Met. The new compound proved to be stable notably longer than the average imaging time in conditions mimicking a biological matrix. Also, it significantly reduced the relaxation times of water, making it a promising candidate as a novel tumour targeting contrast agent for MRI.

## 1. Introduction

Magnetic resonance imaging (MRI) is one of the most important imaging methods due to its non-invasiveness and image quality. The image quality, or contrast, is often improved by the use of contrast agents. Contrast agents are paramagnetic molecules that shorten either longitudinal (*T*_1_) or transverse (*T*_2_) relaxation times, or both, of water protons [1,2]. Most commonly these paramagnetic agents are metal complexes based on gadolinium [3], manganese [4] and iron [5]. Although the use of these agents is common and safe, the risk of releasing toxic metals has raised concerns about the safety of both patients and environment [6]. For instance, the largest group of contrast agents, gadolinium-based contrast agents (GBCAs), may cause nephrogenic systemic fibrosis (NSF) to patients with renal failure [7]. Also, for healthy patients, the repeated use of GBCAs should be avoided [8] due to the deposition of gadolinium in the brain [9]. Moreover, several contrast agents containing iron have been abandoned or discontinued [10]. To overcome these problems, the study of new, metal-free contrast agents has increased rapidly. One of the most attractive research trends has been the study of stable organic radicals, namely nitroxides as a new group of metal-free contrast agents.

Since the 80s, when nitroxides were first mentioned to have contrast enhancement properties [11], the research has continued steadily. The largest drawback of using nitroxides as contrast agents is the fact that these organic radicals often possess only one unpaired electron in contrast to metals, which have seven (Gd), five (Mg), or four (Fe). Therefore, the research has lately focused on macromolecular nitroxide contrast agents [12,13,14,15,16]. This allows inserting several radical units to the contrast agent structure. As the radical centres need to be in contact with the water molecules, linkers are often used to extend the active site. However, this will furthermore enlarge the molecular size. Molecules with large molecular size or strong hydrophilicity cannot cross healthy blood-brain barrier (BBB) [17]. This limits the diagnostics of early stage brain tumours with still intact BBB, which is crucial for most effective treatment [18].

What is common to majority of contrast agents in clinical use is their low specificity and selectivity. In radiopharmaceutical chemistry, target-specificity has been a significant aspect for quite a long time [19]. For example, another important medical imaging method, positron emission tomography (PET), generally applies targeting radiotracers in imaging. Also, nitroxide-based contrast agents have been attached for instance to anti-inflammatory drugs [20], folic acid [21] and glucose [22,23]. Quite recently, we discovered a potential tumour targeting contrast agent that consisted of a nitroxide radical connected with glucose as a targeting unit [23]. The compound comprised an extremely stable radical, 2,2,6,6-tetraethylpiperidinoxide (TEEPO). The purpose of this study was to broaden the scope of the TEEPO-based contrast agents with a new targeting unit. Amino acids, such as methionine (Met), serve as building blocks in protein synthesis, which is strongly associated with cell growth [24]. Due to the increased amino acid uptake in tumours and its common use as a targeting radiotracer in PET, methionine was chosen as the targeting unit in this study. Also, the targeting abilities of methionine have already been studied by preparing targeting gadolinium-based nanoprobes for MRI [25]. This study comprises the synthesis of the new targeting unit and assessment of its stability by EPR spectroscopy as well as relaxation enhancing properties by in vitro NMR spectroscopy and a phantom MRI study.

## 2. Results

### 2.1. Synthesis of the Contrast Agent

**Scheme 1:**
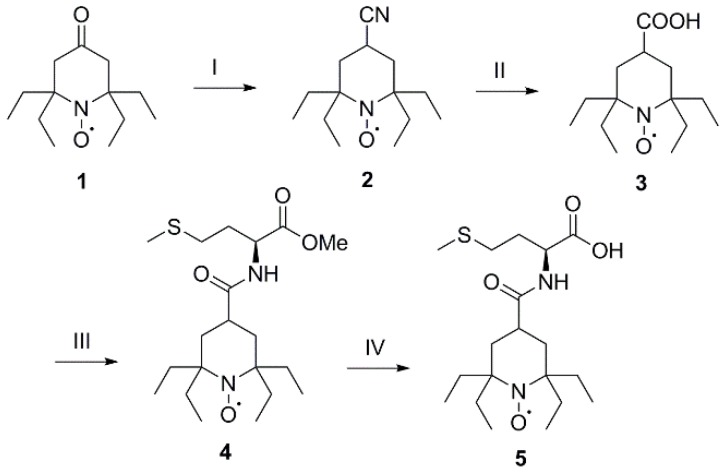
Synthesis of 2,2,6,6-tetraethylpiperidinoxide-methionine (TEEPO-Met). Reagents and conditions: (I) TOSMIC (1.05 eq), *t*-BuOK (2 eq), *t*-butanol, DME, 0 °C, 45 min, rt, 1 h, quant.; (II) Ba(OH)_2_ · 8 H_2_O (3.5 eq), NaOH (1.5 eq), MeOH, H_2_O, 75 °C, 16 h, quant.; (III) DCC (1 eq), HOBt hydrate (1 eq), THF, 0 °C, 20 min; then H-Met-OMe · HCl (1 eq), NMM (1 eq), rt, 24 h, 50%; (IV) 2 N NaOH, MeOH, pH 11, rt, 4 h, 100%; TOSMIC = *p*-Toluenesulfonylmethyl isocyanide, DME = 1,2-Dimethoxyethane, DCC = *N*,*N*’-Dicyclohexylcarbodiimide, HOBt = 1-Hydroxybenzotriazole, H-Met-OMe · HCl = L-Methionine methyl ester hydrochloride, NMM = 4-Methylmorpholine.

The contrast agent 4-[(L-methionine)carbonyl]-2,2,6,6-tetraethylpiperidin-1-oxyl (TEEPO-Met, **5**) was prepared by connecting TEEPO to methionine by an amide bond (Scheme 1). The bonding site of methionine was chosen according to earlier studies [25]. The starting material, 4-oxo-2,2,6,6-tetraethylpiperidin-1-oxyl (4-oxo-TEEPO, **1**) [26], was transformed to 4-carboxy-2,2,6,6-tetraethylpiperidin-1-oxyl (4-COOH-TEEPO, **3**), via 4-carbonitrile-2,2,6,6-tetraethylpiperidin-1-oxyl (4-CN-TEEPO, **2**) adapting a method by Studer et al. [27]. The coupling reaction was performed using *N*,*N*′-dicyclohexylcarbodiimide (DCC) as the activating agent in the presence of hydroxybenzotriazole (HOBt) hydrate and 4-methylmorpholine (NMM) yielding 4-[(L-methionine methyl ester)carbonyl]-2,2,6,6-tetraethylpiperidin-1-oxyl (TEEPO-Met-OMe, **4**) [28]. Methyl ester deprotection of **4** yielded the final product TEEPO-Met (**5**). To compare the stability of TEEPO-Met to a less sterically shielded radical; 4-[(L-methionine)carbonyl]-2,2,6,6-tetramethylpiperidin-1-oxyl (TEMPO-Met, **7**) was prepared. The synthesis followed the same procedure as for **5**.

### 2.2. EPR Studies

**Figure 1:**
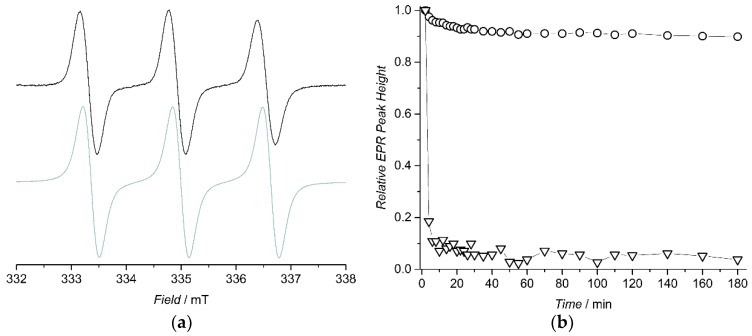
(**a**) Solution-state X-band electron paramagnetic resonance (EPR) spectrum of **5** (black) at 291 K in phosphate buffered solution (PBS) without ascorbic acid and simulated EPR spectrum of **5** (green); (**b**) Reduction profiles of **5** (circle) and **7** (triangle) in 25 mM PBS (pH 7.4.) with 10 vol % of DMSO at 291 K (line to guide the eye). Concentrations of 0.2 mM and 4 mM were used for nitroxide and ascorbic acid, respectively.

To confirm the radical nature of **5** and **7**, their X-band EPR spectra were first measured in a pure phosphate buffered solution (PBS) with 10 vol % of dimethyl sulfoxide (DMSO) and without a reduction agent. As seen in Figure 1a, the EPR spectrum of **5** shows a triplet pattern, arising from ^14^N hyperfine splitting, with an isotropic hyperfine coupling constant (IHCC) of 46 MHz and g_iso_-value of 2.0057. A similar spectrum and parameters (a_N_ = 49 MHz and g_iso_ = 2.0055) were also obtained for **7** (Figure S2). Observed peak patterns, IHCCs and g_iso_-values for **5** and **7** are in line with previously reported values for analogous nitroxide radicals [29,30].

To investigate the reduction stability of synthesized nitroxide radicals in conditions mimicking biological matrix, their PBSs were treated with a 20-fold molar excess of ascorbic acid and the decay of high-field EPR peak height was monitored with respect to time at 291 K. The TEEPO-Met contrast agent **5** showed very good resistance towards reduction of ascorbic acid; after three hours only 10% of radical was consumed in reductive conditions (Figure 1b). In sharp contrast to **5**, the less sterically protected nitroxide radical **7** was almost fully reduced to diamagnetic hydroxylamine in 10 min and after 40 min only a very weak signal was observed in the EPR spectrum.

### 2.3. NMR Relaxometric Studies In Vitro

**Figure 2:**
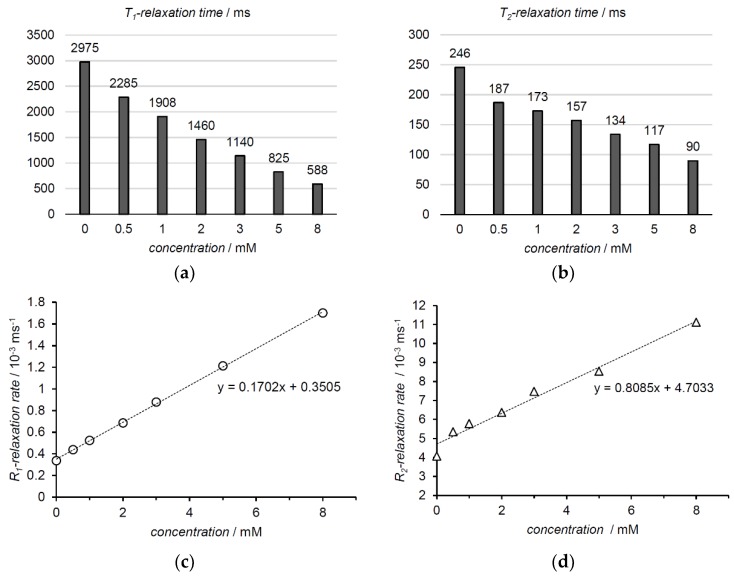
The water proton relaxation times in human blood plasma were measured at 37 °C in 11.7 T magnetic field (500 MHz ^1^H-frequency). The relaxation rates and the slopes representing relaxivities were calculated from relaxation times and their changes in different concentrations (**a**) *T*_1_-relaxation times at different nitroxide concentrations; (**b**) *T*_2_-relaxation times at different nitroxide concentrations; (**c**) *R*_1_-relaxation rate as a function of nitroxide concentration and the slope representing r_1_-relaxivity (linear fit and corresponding equation); (**d**) *R*_2_-relaxation rate as a function of nitroxide concentration and the slope representing *r*_2_-relaxivity (linear fit and corresponding equation).

In 11.7 T (500 MHz) ^1^H NMR preliminary relaxometric studies, water relaxation times in human blood plasma at 37 °C were studied as a function of nitroxide concentration. The graph in Figure 2a shows a decrease in *T*_1_-relaxation time as the concentration of the contrast agent rises. A similar trend was observed for *T*_2_-relaxation time (Figure 2b). Inverses of the *T*_1_- and *T*_2_-relaxation times gave *R*_1_- and *R*_2_-relaxation rates, respectively. Their presentation as a function of concentration produced a linear regression equation (Figure 2c,d). The slopes of the functions defined relaxivities *r*_1_ and *r*_2_ for the nitroxide, resulting in 0.17 mM^−1^ s^−1^ and 0.81 mM^−1^ s^−1^, respectively. The relaxivity ratio *r*_2_/*r*_1_ at 11.7 T was 4.8.

### 2.4. Phantom MRI Studies

**Figure 3:**
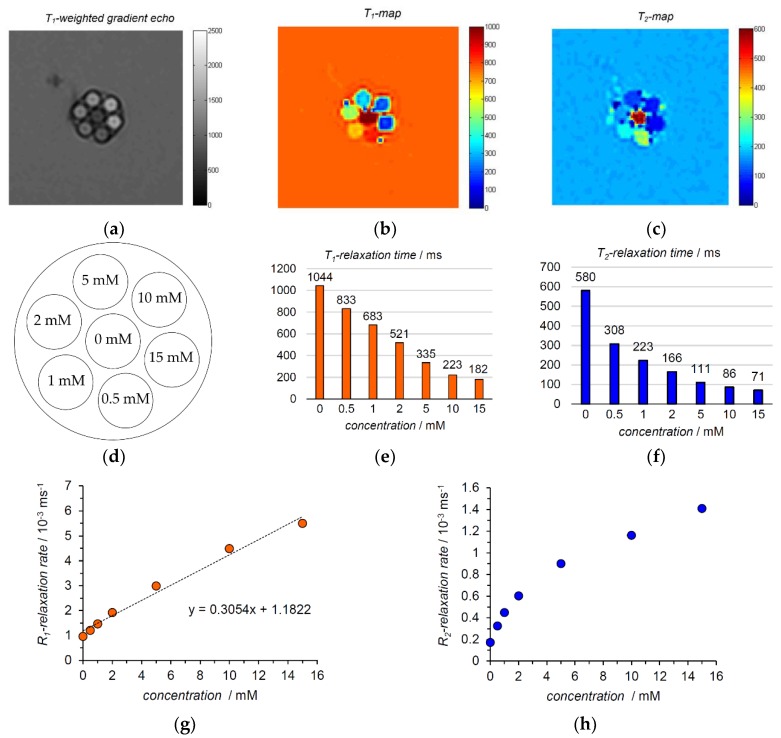
A small sized phantom (approximately 15 mm × 15 mm, tube inner diameter 4 mm) imaged in 1.5 T clinical MRI instrument: (**a**) *T*_1_-weighted FLASH image. Dark pixels visible in the centre of each tube originate from truncation artefact. This is a result of utilized low imaging resolution (0.8 mm/pixel) compared to small diameter of MRI-visible sample within the tube (4 mm) surrounded by MRI-invisible tube walls.; (**b**) *T*_1_-map, inversion recovery; (**c**) *T*_2_-map, multiecho; (**d**) composition of the phantom with radical concentration values, mM; (**e**) *T*_1_-relaxation times obtained from the *T*_1_-map; (**f**) *T*_2_-relaxation times obtained from the *T*_2_-map; (**g**) *R*_1_-relaxation rate as a function of concentration and *r*_1_-relaxivity from the slope of the function (linear fit and corresponding equation); (**h**) *R*_2_-relaxation rate as a function of concentration.

The results of the 1.5 T phantom MRI study at ambient temperature (22 °C) indicated improved contrast between different samples with varying nitroxide concentrations. This is visible in *T*_1_-weighted fast low angle shot (FLASH) image (Figure 3a). Also inversion-recovery *T*_1_- and multiecho *T*_2_-maps emphasized the differences in relaxation times (Figure 3b,c). In accordance to the 11.7 T NMR data, the *T*_1_- and *T*_2_-relaxation times decreased as the nitroxide concentration went up (Figure 3e,f). The *R*_1_-relaxation rate increased linearly as a function of concentration (Figure 3f). The slope of the linear function gave relaxivity *r*_1_ a value of 0.31 mM^−1^ s^−1^. The *R*_2_-relaxation rate also increased with the concentration but in a non-linear manner (Figure 3h).

## 3. Discussion

The purpose of the study was to synthesize a new radical MRI contrast agent carrying an amino acid functionality with potential tumour targeting properties. The study also assessed the properties of the agent concerning its stability and relaxation enhancing properties. The stable nitroxide, TEEPO, was selected as a radical moiety, as it proved an excellent choice in our previous study concerning its stability and relaxation properties [23]. TEEPO was successfully attached to the methionine amino acid which has been shown to increase cellular uptake of various tracers in tumours for instance in PET [31]. The targeting effect of methionine and its accumulation to tumour is to be assessed in future with in vivo MRI tests.

One of the most prominent issues concerning radicals is their stability. The main threat to the nitroxide stability is its reduction to corresponding hydroxylamine by natural reductants, enzymes or antioxidants in human body. The most prevailing natural reductant is ascorbic acid and it is commonly used in studying nitroxide stability with EPR-spectroscopy as the in vitro results correspond well the lifetime of the radicals in vivo [32]. In this study, the radical samples mimicking biological matrix were composed of phosphate buffered solution corresponding the pH of blood (7.4) and the reduction of the radical was observed as a loss of EPR signal. The results revealed the high stability of the TEEPO-Met especially in contrast to TEMPO-Met, which was almost completely reduced in ten minutes. The results are in accordance with studies of the plain radical moieties [29,30,33]. The stability of nitroxide radicals can be acknowledged mainly to the steric hindrance provided by the substituents adjacent to the radical centre. First of all, the steric hindrance prevents disproportionation of two nitroxides and secondly the access of reactive species to the radical centre is blocked. The effect of the substituent size is clearly seen also in our results as the bulkier ethyl side groups of TEEPO increased the stability drastically compared to the methyl side groups of TEMPO.

The relaxation enhancement abilities of TEEPO-Met were assessed by a relaxometric in vitro NMR study at 11.7 T. Adding contrast agent to blood plasma samples showed a considerable decrease in both *T*_1_- and *T*_2_-relaxation times of the water proton signal. This phenomenon would appear as enhanced contrast between diseased and healthy tissue, thus, improving image quality, provided that contrast agent accumulates in the diseased tissue. The *T*_1_-relaxation time halved at 2 mM solution and further dropped to one third as the concentration reached 5 mM. The relaxation rate increased linearly with the concentration, implicating that the contrast agent shows no aggregation [12]. The relaxation enhancing properties were also evaluated by a phantom MRI study, performed with a 1.5 T MRI scanner in active clinical use. The small size of the phantom caused some restrictions to the image quality as it was imaged with a human sized scanner and head coil. Nevertheless, the results showed a clear contrast between different concentrations. According to these results, TEEPO-Met will affect the relaxation rates and thus the contrast, both in pre-clinical (11.7 T) and clinical (1.5 T) systems.

The obtained *r*_2_/*r*_1_ value 4.8 at 11.7 T magnetic field suggests that TEEPO-Met could function as a *T*_1_-*T*_2_ dual mode contrast agent at high field strengths [34]. While the *r*_2_/*r*_1_ ratio at 11.7 T was relatively high, Figure 3a however displays decent contrast in the 1.5 T *T*_1_-weighted gradient echo image. A very rough estimate for *r*_2_ from 1.5 T data was obtained by performing a linear regression to the non-linear data shown in Figure 3h. This resulted in approximate value of 0.77 mM^−1^ s^−1^ for *r*_2_, leading to the *r*_2_/*r*_1_ ratio value 2.5 at 1.5 T. This is in accordance with the *r*_2_/*r*_1_ ratio value for TEEPO-Glc at 1.5 T (1.7; calculated from the *T*_1_- and *T*_2_-relaxation time data published earlier) [23].

The relaxivities of the synthesized TEEPO-Met correspond well the ones calculated for monoradicals in general [12,35]. However, they are relatively low compared to commonly used GBCAs (*r*_1_ ≈ 3.6–5.2 mM^−1^ s^−1^ at 37 °C in 1.5 T magnetic field [36]), due to the seven unpaired electrons in gadolinium compared to one in TEEPO-Met. According to our hypothesis the possible targeting effect should overcome this flaw as the specificity toward tumour tissue is increased. Future studies will reveal the targeting properties as well as the BBB crossing ability of the agent.

In conclusion, we synthesized a fully organic, metal-free radical contrast agent candidate for MRI with excellent stability and good relaxation enhancing properties. In addition, this contrast agent is potentially tumour-accumulating, which is expected to have a substantial impact on established MRI-based diagnostics.

## 4. Materials and Methods

### 4.1. General Experimental Procedures

Blood plasma used in the in vitro NMR and MRI studies was obtained from the Finnish Red Cross Blood Service. All chemicals were acquired from commercial sources. Solvents were dried by standard methods; dry dimethoxyethane (DME) was obtained with sodium and *tert*-butyl alcohol (*t*-BuOH) by distillation over activated 4 Å molecular sieves. Potassium *tert*-butoxide (*t*-BuOK) was purified by sublimation.

The NMR spectra were recorded with a Varian Unity Inova (Varian Inc., Palo Alto, CA, USA) 500 NMR-spectrometer (500 MHz ^1^H-frequency, 11.7 T). For the analysis, the radical compounds **2**–**7** were reduced to corresponding hydroxylamines by adding excess of hydrazobenzene to the samples. Hydrazobenzene and its oxidized form are visible at 6.5–8 ppm in ^1^H-spectra and at 110–155 ppm in ^13^C-spectra. The high-resolution MS spectra of **2**–**7** were obtained with a Bruker Micro TOF (Bruker Daltonics GmbH, Bremen, Germany) with electron spray ionization (ESI). The EPR spectra of **5** and **7** were scanned with a Magnettech MiniScope MS 200 spectrometer (Magnettech, Berlin, Germany) using DPPH (2,2-diphenyl-1-picrylhydrazyl) as a standard.

### 4.2. Synthesis of the Contrast Agent

*4-Carbonitrile-2,2,6,6-Tetraethylpiperidin-1-Oxyl* (**2**): To a solution of 4-oxo-TEEPO **1** (502.9 mg; 2.22 mmol) in 30 mL of DME was added 400 mg (2.05 mmol) of TOSMIC and the reaction mixture was brought to 0 °C. A solution containing 500 mg (4.46 mmol) of *t*-BuOK in 4 mL of DME and 4 mL of *t*-BuOH was added slowly to the reaction mixture. The reaction mixture was stirred under argon at 0 °C for 45 min, then brought to room temperature and further stirred for one hour. To the mixture, 40 mL of H_2_O was added and the solution was extracted three times with Et_2_O (20 mL). The organic layers were combined, dried over MgSO_4_ and evaporated. The reaction yielded a quantitative amount (0.5322 g) of a red solid product, which was used without further purification. ^1^H NMR (CDCl_3_, hydrazobenzene, 500 MHz) δ 2.72 (1 H, tt, ^3^*J* = 12.8 Hz, ^3^*J* = 3.3 Hz, CH), 1.91–1.98 (2 H, qd, ^2^*J* = −14.5 Hz, ^3^*J* = 7.3 Hz, CH_2_), 1.79–1.82 (2 H, m, CH_2_), 1.66–1.73 (2 H, dq, ^2^*J* = −14.4 Hz, ^3^*J* = 7.2 Hz, CH_2_), 1.62–1.67 (2 H, m, CH_2_), 1.49–1.56 (2 H, dq, ^2^*J* = −14.5 Hz, ^3^*J* = 7.3 Hz, CH_2_), 1.27–1.35 (2 H, dq, ^2^*J* = −14.4 Hz, ^3^*J* = 7.2 Hz, CH_2_), 0.85 (6 H, t, ^3^*J* = 7.2 Hz, CH_3_), 0.83 (6 H, t, ^3^*J* = 7.3 Hz, CH_3_); ^13^C NMR (CDCl_3_, hydrazobenzene, 126 MHz) δ 122.7 (CN), 62.4 (C), 33.6 (CH_2_), 29.6 (CH_2_), 25.6 (CH_2_), 20.2 (CH), 9.6 (CH_3_), 8.1 (CH_3_); ESI-MS *m*/*z* 260.1868 [M + Na]^+^ (calcd. for C_14_H_25_N_2_O, 260.1859 [M + Na]^+^).

*4-Carboxy-2,2,6,6-Tetraethylpiperidin-1-Oxyl* (**3**): The 4-CN-TEEPO **2** (527.4 mg; 2.22 mmol) was dissolved in MeOH (6 mL). To the mixture, Ba(OH)_2_ · 8 H_2_O (2452.9 mg; 7.78 mmol) and NaOH (133.6 mg; 3.34 mmol) in 20 mL of H_2_O was added. The mixture was stirred at 75 °C overnight. The aqueous mixture was extracted with CHCl_3_ (20 mL), acidified to pH 2 with 10% HCl and extracted three times with 20 mL of CHCl_3_. The organic residue was dried over MgSO_4_ and evaporated to dryness. The product, 4-COOH-TEEPO (606.6 mg), was collected with a quantitative yield as a red oil and was used as such. ^1^H NMR (CD_3_OD, hydrazobenzene, 500 MHz) δ 2.57 (1 H, tt, ^3^*J* = 12.7 Hz, ^3^*J* = 3.1 Hz, CH), 1.94–2.01 (2 H, dq, ^2^*J* = −14.6 Hz, ^3^*J* = 7.5 Hz, CH_2_), 1.69–1.72 (2 H, m, CH_2_), 1.62–1.67 (2 H, dq, ^2^*J* = −14.1 Hz, ^3^*J* = 7.2 Hz, CH_2_), 1.62–1.67 (2 H, dq, ^2^*J* = −14.6 Hz, ^3^*J* = 7.5 Hz, CH_2_), 1.50 (2 H, dd, ^3^*J* = 12.7 Hz, ^2^*J* = −12.7 Hz, CH_2_), 1.28–1.35 (2 H, dq, ^2^*J* = −14.1 Hz, ^3^*J* = 7.2 Hz, CH_2_), 0.90 (6 H, t, ^3^*J* = 7.2 Hz, CH_3_), 0.84 (6 H, t, ^3^*J* = 7.5 Hz, CH_3_); ^13^C NMR (CD_3_OD, hydrazobenzene, 126 MHz) δ 180.2 (COOH), 63.7 (C), 35,0 (CH), 34.1 (CH_2_), 30.9 (CH_2_), 26.8 (CH_2_), 10.0 (CH_3_), 8.2 (CH_3_); ESI-MS *m*/*z* 279.1782 [M + Na]^+^ (calcd. for C_14_H_26_NO_3_, 279.1805 [M + Na]^+^).

*4-[(L-Methionine Methyl Ester)Carbonyl]-2,2,6,6-Tetraethylpiperidin-1-Oxyl* (**4**): A solution of 4-COOH-TEEPO **3** (606.6 mg; 2.46 mmol) in 25 mL of anhydrous THF was brought to 0 °C. To the reaction mixture, DCC (456.5 mg; 2.21 mmol) and HOBt hydrate (272.8 mg) were added and the mixture was stirred at 0 °C for 15 min. Then, H-Met-OMe · HCl (399.3 mg; 2.00 mmol) and NMM (0.2 mL) were added and the mixture was brought to room temperature and stirred under argon overnight. After completion of the reaction, the mixture was filtered and diluted with EtOAc (20 mL). The organic solution was washed with 5% NaHCO_3_ (20 mL), brine (20 mL), 5% KHSO_4_ (20 mL) and again with brine (20 mL). The organic phase was dried with Na_2_SO_4_, filtered and evaporated. The crude product was purified by silica gel column chromatography using 3:1 hexane/EtOAc mixture as eluent. The product was collected as an orange solid (404.1 mg; 50%). ^1^H NMR (CDCl_3_, hydrazobenzene, 500 MHz) δ 6.24 (1 H, br, NH), 4.69–4.73 (1 H, m, CH), 3.77 (3 H, s, CH_3_), 2.52 (2H, t, ^3^*J* = Hz, CH_2_), 2.42–2.49 (1 H, m, CH), 2.14–2.21 (1 H, m, CH_2_), 2.10 (3 H, s, CH_3_), 1.98–2.04 (1 H, m, CH_2_), 1.94–2.01 (2 H, dq, ^2^*J* = −14.9 Hz, ^3^*J* = 7.5 Hz, CH_2_), 1.67–1.72 (2 H, m, CH_2_), 1.63–1.67 (2 H, m, CH_2_), 1.55–1.63 (4 H, m, CH_2_), 1.27–1.34 (2 H, dq, ^2^*J* = −14.1 Hz, ^3^*J* = 7.3 Hz, CH_2_), 0.86 (6 H, t, ^3^*J* = 7.3 Hz, CH_3_), 0.84 (6 H, t, ^3^*J* = 7.5 Hz, CH_3_); ^13^C NMR (CDCl_3_, hydrazobenzene, 126 MHz) δ 175.4 (CO), 172.7 (COO), 62.4 (C), 52.6 (OCH_3_), 51.5 (CH), 35.5 (CH), 33.4 (CH_2_), 33.1 (CH_2_), 31.6 (CH_2_), 30.2 (SCH_2_), 29.9 (CH_2_), 25.9 (CH_2_), 15.6 (SCH_3_), 9.7 (CH_3_), 8.2 (CH_3_); ESI-MS *m*/*z* 424.2391 [M + Na]^+^ (calcd. for C_20_H_37_N_2_O_4_S, 424.2366 [M + Na]^+^).

*4-[(L-Methionine)Carbonyl]-2,2,6,6-Tetraethylpiperidin-1-Oxyl* (**5**): A mixture of the methyl ester protected TEEPO-Met **4** (389.2 mg; 9.69 mmol) in 5 mL of MeOH and 5 mL of 2 M NaOH solution was stirred at room temperature for 2 h. The pH of the mixture was adjusted to 7 with 10% HCl solution. Methanol was evaporated and the residue was adjusted to pH 2 with 2% HCl. The acidic water phase was extracted two times with EtOAc. The organic layer was washed with brine, dried over Na_2_SO_4_ and evaporated to dryness. The reaction yielded pure final product as an orange powder (374.7 mg; 100%). ^1^H NMR (DMSO, hydrazobenzene, 500 MHz) δ 8.08 (1H, d, ^3^*J* = 7.8 Hz, NH), 4.25–4.29 (1 H, m, CH), 2.52–2.54 (1H, m, CH), 2.41–2.47 (2 H, m, CH_2_), 2.04 (3 H, s, CH_3_), 1.95–1.99 (1 H, m, CH_2_), 1.85–1.95 (2 H, m, CH_2_), 1.81–1.85 (1 H, m, CH_2_), 1.52–1.59 (4 H, m, CH_2_), 1.34–1.44 (4 H, m, CH_2_), 1.10–1.19 (2 H, m, CH_2_), 0.82 (6 H, t, ^3^*J* = 7.7 Hz, CH_3_), 0.77 (6 H, t, ^3^*J* = 7.1 Hz, CH_3_); ^13^C NMR (DMSO, hydrazobenzene, 126 MHz) δ 175.2 (CO), 173.4 (COO), 61.2 (C), 50.7 (CH), 33.7 (CH), 32.9 (CH_2_), 32.6 (CH_2_), 30.7 (CH_2_), 29.8 (CH_2_), 29.5 (CH_2_), 25.3 (CH_2_), 14.6 (CH_3_), 9.8 (CH_3_), 7.9 (CH_3_); ESI-MS *m*/*z* 386.2242 [M]^−^ (calcd. for C_19_H_35_N_2_O_4_S, 386.2234 [M]^−^).

*4-[(L-Methionine Methyl ester)Carbonyl]-2,2,6,6-Tetramethylpiperidin-1-Oxyl* (**6**): The reaction was performed using the same procedure as for **4**. Purification 2:1 hexane/EtOAc, yield 64%, 0.2812 g. ^1^H NMR (CDCl_3_, hydrazobenzene, 500 MHz) δ 6.24 (1 H, d, ^3^*J* = 7.6 Hz, NH), 4.70–4.74 (1 H, m, CH), 3.76 (3 H, s, CH_3_), 2.53–2.56 (1 H, m, CH), 2.46–2.53 (2 H, m, CH_2_), 2.14–2.21 (1 H, m, CH_2_), 2.10 (3 H, s, CH_3_), 1.95–2.03 (1 H, m, CH_2_), 1.65–1.75 (4 H, m, CH_2_), 1.20 (3 H, s, CH_3_), 1.20 (3 H, s, CH_3_), 1.15 (3 H, s, CH_3_), 1.14 (3 H, s, CH_3_); ^13^C NMR (CDCl_3_, hydrazobenzene, 126 MHz) δ 174.9 (CO), 172.8 (COO), 58.6 (C), 52.7 (CH_3_), 51.5 (CH), 42.3 (CH_2_), 42.1 (CH_2_), 36.5 (CH), 32.3 (CH_3_), 32.3 (CH_3_), 31.8 (CH_2_), 30.1 (CH_2_), 19.7 (CH_3_), 19.6 (CH_3_), 15.6 (CH_3_); ESI-MS *m*/*z* 368.1725 [M + Na]^+^ (calcd. for C_16_H_29_N_2_O_4_S, 368.1740 [M + Na]^+^).

*4-[(L-Methionine)Carbonyl]-2,2,6,6-Tetramethylpiperidin-1-Oxyl* (**7**): The reaction was performed using the same procedure as for **5**. Yield 82%, 0.1620 g. ^1^H NMR (DMSO, hydrazobenzene, 500 MHz) δ 8.08 (1H, d, ^3^*J* = 7.9 Hz, NH), 4.25–4.30 (1 H, m, CH), 2.61 (1 H, tt, ^3^*J* = 12.0 Hz, ^3^*J* = 3.9 Hz, CH), 2.42–2.52 (2 H, m, CH_2_), 2.04 (3 H, s, CH_3_), 1.90–1.99 (1 H, m, CH_2_), 1.80–1.87 (1 H, m, CH_2_), 1.44–1.56 (4 H, m, CH_2_), 1.07 (3 H, s, CH_3_), 1.06 (6 H, s, CH_3_), 1.05 (3 H, s, CH_3_); ^13^C NMR (DMSO, hydrazobenzene, 126 MHz) δ 174.6 (CO), 173.7 (COO), 57.6 (C), 50.8 (CH), 41,9 (CH_2_), 41.8 (CH_2_), 34.7 (CH), 32.3 (CH_3_), 30.8 (CH_2_), 29.7 (CH_2_), 19.6 (CH_3_), 14.6 (CH_3_); ESI-MS *m*/*z* 330.1627 [M]^−^ (calcd. for C_15_H_27_N_2_O_4_S, 330.1608 [M]^−^).

### 4.3. EPR Study

The EPR samples for stability study were prepared by combining equimolar amounts of two phosphate buffered solutions (PBS), the first containing the radical and the second a 20-fold molar excess of ascorbic acid. The solution containing the radical was prepared using appropriate amounts of monosodium phosphate hydrate and disodium phosphate dihydrate. The solution was doped with diethylenetriaminepentaacetic acid (DTPA, 0.1 mM) and pH was adjusted to 7.4 with 2 M NaOH solution. The radical was dissolved to an appropriate amount of DMSO and diluted with PBS so that a 25 mM PBS solution with 10 vol % of DMSO and was obtained. The ascorbic acid solution was prepared analogously but instead of the radical ascorbic acid was added. Concentrations of 0.2 mM 4 mM of were used for nitroxide and ascorbic acid, respectively. The solution state EPR spectra of **5** and **7** were obtained in PBS without ascorbic acid.

X-band EPR spectra were recorded on Magnettech MiniScope MS 200 spectrometer (Magnettech, Berlin, Germany) at 291 K. The following set of parameters were used in measurements for **5**: ν ≈ 9.4 GHz, centre field = 3350.47 mT, spectrum width = 70 mT, modulation amplitude ≈ 0.1 mT and microwave attenuation = 15 dB. For **7** centre field and microwave attenuation were set for 3349.64 mT and 18 dB, respectively, otherwise same parameters were used than for **5**. The obtained experimental g_iso_ values of 2.0047 and 2.0045 were corrected with respect to the DPPH powder (2.0036) to give g_iso_ values of 2.0057 and 2.0055 for **5** and **7**, respectively.

EPR spectra simulations for the solution spectra of **5** and **7** were performed with the EasySpin toolbox for Matlab (The MathWorks Inc., Natick, MA, USA) [37]. The microwave frequency, modulation amplitude, g-value and centre field used in simulations were obtained from experimental settings. For **5**, the best match between the experimental and simulated spectrum in simulations was obtained by using Gaussian peak-to-peak linewidth 0.20 mT, Lorentzian peak-to-peak linewidth 0.14 mT and ^14^N isotropic hyperfine splitting constant 45.99 MHz, whereas for **7** the same parameters were 0.13 mT, 0.06 mT and 48.75 mT.

### 4.4. In Vitro NMR Study

The relaxation times were measured as a function of concentration of the contrast agents. The concentration was increased in situ by adding appropriate amount of stock solution to the sample. The stock solution was prepared by dissolving 4.3 mg of TEEPO-Met into 50 μL of DMSO and adding 450 μL of blood plasma resulting a 22.2 mM solution. The initial sample was prepared by mixing 60 μL of D_2_O, 60 μL of DMSO and 480 μL of blood plasma. After measuring the *T*_1_- and *T*_2_-relaxation times, an appropriate amount of the stock solution was added and the measurements recorded. This was repeated so that the relaxation times for concentrations of 0, 0.5, 1, 2, 3, 5 and 8 mM were achieved.

The *T*_1_-relaxation times were measured using a saturation-recovery experiment, where the magnetization is initially destroyed by two pulse-gradient elements followed by a recovery delay and final excitation pulse. In the experiment 14 spectra were recorded with recovery delays of 3, 10, 20, 40, 100, 150, 200, 300, 400, 800, 1000, 2000, 4000 and 6000 ms. The acquisition time and relaxation delay were 2000 and 4000 ms, respectively. The *T*_2_-relaxation times were measured utilizing a CPMG experiment (Carr-Purcell-Meiboom-Gill spin echo pulse train) [38,39]. In order to determine *T*_2_-values, seven spectra with different CPMG-durations (20, 40, 80, 100, 200, 300, 500 ms) were measured. The individual 180° pulses in the CPMG pulse-train were flanked by 5 ms delay. The acquisition time of 2000 ms and relaxation delay of 12,000 ms were used.

The study was performed with a Varian Unity Inova (Varian Inc., Palo Alto, CA, USA) 500 NMR-spectrometer (500 MHz ^1^H-frequency, 11.7 T) at 37 °C. The spectrometer was equipped with 5 mm pulsed field gradient inverse detection dual ^1^H-X probe head capable of delivering z-axis gradient amplitudes up to 20 G/cm. The X-coil is tunable from ^15^N to ^31^P frequencies. The spectrometer operating software was Vnmr 6.1C (Varian Inc., Palo Alto, CA, USA). The *T*_1_- and *T*_2_-relaxation times were calculated using the fitting methods provided in the spectrometer operating software Vnmr 6.1C.

### 4.5. Phantom MRI Study

The MRI-phantom consisted seven samples containing TEEPO-Met in concentrations of 0, 0.5, 1, 2, 5, 10 and 15 mM. The contrast agent was dissolved in 10% DMSO/blood plasma solution and the phantom was sunk into a 1 L container filled with 0.1 mM MnCl_2_/ water solution containing 0.4 wt-% of NaCl. The samples were prepared in 4 mm inner diameter tubes with 600 μL of sample solution. A Teflon holder assuring their parallel position held the sample tubes together.

The MRI-phantom was imaged at approximately 22 °C (ambient temperature) with a 1.5 T Siemens MAGNETOM Avantofit (Siemens Medical Solutions, Erlangen, Germany) clinical MRI scanner using 20-channel head/neck receiving coil for signal detection. The *T*_1_-weighted MRI-image was obtained with a fast low angle shot (FLASH) sequence. The slice thickness was set to 5 mm, field of view (FOV) 200 × 200 mm and matrix size 256 × 256. The repetition time (TR) was 200 ms, echo time (TE) 4.76 ms and the flip angle 90°. The *T*_1_-mapping was performed with an inversion recovery experiment. In the experiment, slice thickness was 5 mm, FOV 200 × 200 mm and matrix size 192 × 192. Inversion times were 150, 300, 450, 600, 750 and 900 ms while TR and TE were 2000 and 30 ms, respectively. The *T*_2_-mapping was performed with a multi-echo spin-echo sequence. The TE had 32 different values ranging from 25 ms to 800 ms with 25 ms increments. The TR was 1000 ms, the slice thickness 5 mm, FOV 250 × 250 mm and the matrix size 256 × 256. The relaxation time maps were calculated with an in-house written MatLab script (The MathWorks Inc., Natick, MA, USA).

## Supplementary Materials

The following are available online. Figure S1: Solution-state X-band EPR spectrum of 4-[(L-Methionine)carbonyl]-2,2,6,6-tetraethylpiperidin-1-oxyl (TEEPO-Met, **5**) in PBS (black) and simulated EPR spectrum (green), Figure S2: Solution-state X-band EPR spectrum of 4-[(L-Methionine)carbonyl]-2,2,6,6-tetramethylpiperidin-1-oxyl (TEMPO-Met, **7**) in PBS (black) and simulated EPR spectrum (green), Figure S3: ^1^H spectrum of 4-Carbonitrile-2,2,6,6-tetraethylpiperidin-1-oxyl (4-CN-TEEPO, **2**) in CDCl_3_, Figure S4: ^13^C spectrum of 4-Carbonitrile-2,2,6,6-tetraethylpiperidin-1-oxyl ((4-CN-TEEPO, **2**) in CDCl_3_, Figure S5: ^1^H spectrum of 4-Carboxy-2,2,6,6-tetraethylpiperidin-1-oxyl (4-COOH-TEEPO, **3**) in CD_3_OD, Figure S6: ^13^C spectrum of 4-Carboxy-2,2,6,6-tetraethylpiperidin-1-oxyl (4-COOH-TEEPO, **3**) in CD_3_OD, Figure S7: ^1^H spectrum of 4-[(L-Methionine methyl ester)carbonyl]-2,2,6,6-tetraethylpiperidin-1-oxyl (TEEPO-Met-OMe, **4**) in CDCl_3_, Figure S8: ^13^C spectrum of 4-[(L-Methionine methyl ester)carbonyl]-2,2,6,6-tetraethylpiperidin-1-oxyl (TEEPO-Met-OMe, **4**) in CDCl_3_, Figure S9: ^1^H spectrum of 4-[(L-Methionine)carbonyl]-2,2,6,6-tetraethylpiperidin-1-oxyl (TEEPO-Met, **5**) in DMSO, Figure S10: ^13^C spectrum of 4-[(L-Methionine)carbonyl]-2,2,6,6-tetraethylpiperidin-1-oxyl (TEEPO-Met, **5**) in DMSO, Figure S11: ^1^H spectrum of 4-[(L-Methionine methyl ester)carbonyl]-2,2,6,6-tetramethylpiperidin-1-oxyl (TEMPO-Met-OMe, **6**) in CDCl_3_, Figure S12: 13C spectrum of 4-[(L-Methionine methyl ester)carbonyl]-2,2,6,6-tetramethylpiperidin-1-oxyl (TEMPO-Met-OMe, **6**) in CDCl_3_, Figure S13: 1H spectrum of 4-[(L-Methionine)carbonyl]-2,2,6,6-tetramethylpiperidin-1-oxyl (TEMPO-Met, **7**) in DMSO, Figure S14: ^13^C spectrum of 4-[(L-Methionine)carbonyl]-2,2,6,6-tetramethylpiperidin-1-oxyl (TEMPO-Met, **7**) in DMSO.
