# Supplementary material for: Synthesis, Stability and Relaxivity of TEEPO-Met: An Organic Radical as a Potential Tumour Targeting Contrast Agent for Magnetic Resonance Imaging
Source: Molecules. 2018 Apr 27;23(5):1034. doi: 10.3390/molecules23051034 (PMC6102570; doi:10.3390/molecules23051034)
Supplement: Supplementary file 1 [file molecules-23-01034-s001.pdf]

Supplementary Material

Synthesis, Stability and Relaxivity of TEEPO-Met: An Organic Radical as a Potential Tumour Targeting Contrast Agent for Magnetic Resonance Imaging

Maiju Soikkeli [1], Kaisa Horkka [1,2], Jani O. Moilanen [3], Marjut Timonen [4], Jari Kavakka [1,5] and Sami Heikkinen [1,*]

[1] Department of Chemistry, University of Helsinki, P.O. Box 55, 00014 Helsinki, Finland; maiju-lotta.soikkeli@helsinki.fi (M.S.); kaisa.horkka@ki.se (K.H.); jari.kavakka@storaenso.com (J.K.)
[2] Department of Clinical Neuroscience, Karolinska Institutet, S-17176 Stockholm, Sweden
[3] Department of Chemistry, Nanoscience Centre, University of Jyväskylä, P.O. Box 35, 40014 Jyväskylä, Finland; jani.o.moilanen@jyu.fi
[4] HUS Helsinki Medical Imaging Center, Radiology, University of Helsinki and Helsinki University Hospital, P.O. Box 340, 00029 HUS, Helsinki, Finland; marjut.timonen@hus.fi
[5] Stora Enso, Innovation Centre for Biomaterials, Fannys väg 1, S-13154 Nacka, Sweden
* Correspondence: sami.heikkinen@helsinki.fi; Tel: +358-2941-50368

Table of contents

EPR spectra of compounds **5** and **7** (Fig S1-S2) S2

NMR spectra of compounds **2**-**7** (Fig S3-S14) S3

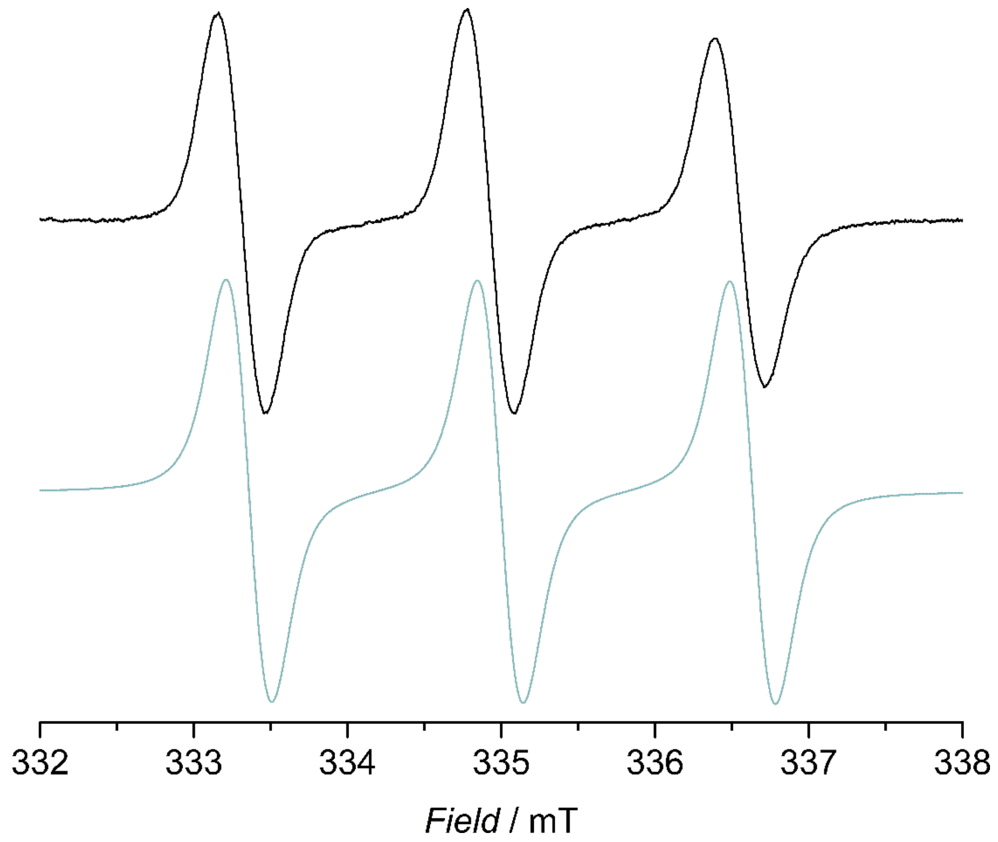

Figure S1. Solution-state X-band EPR spectrum of 4-[(L-Methionine)carbonyl]-2,2,6,6-tetraethylpiperidin-1-oxyl (TEEPO-Met, **5**) in PBS (black) and simulated EPR spectrum (green).

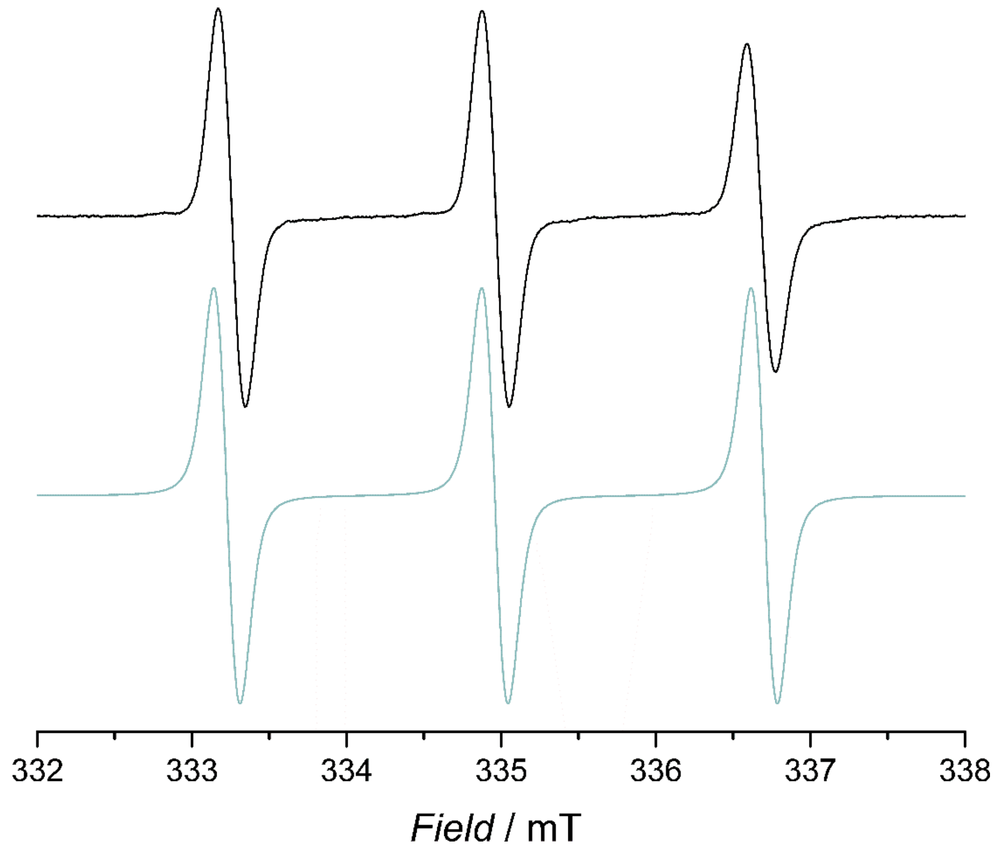

Figure S2. Solution-state X-band EPR spectrum of 4-[(L-Methionine)carbonyl]-2,2,6,6-tetramethylpiperidin-1-oxyl (TEMPO-Met, **7**) in PBS (black) and simulated EPR spectrum (green).

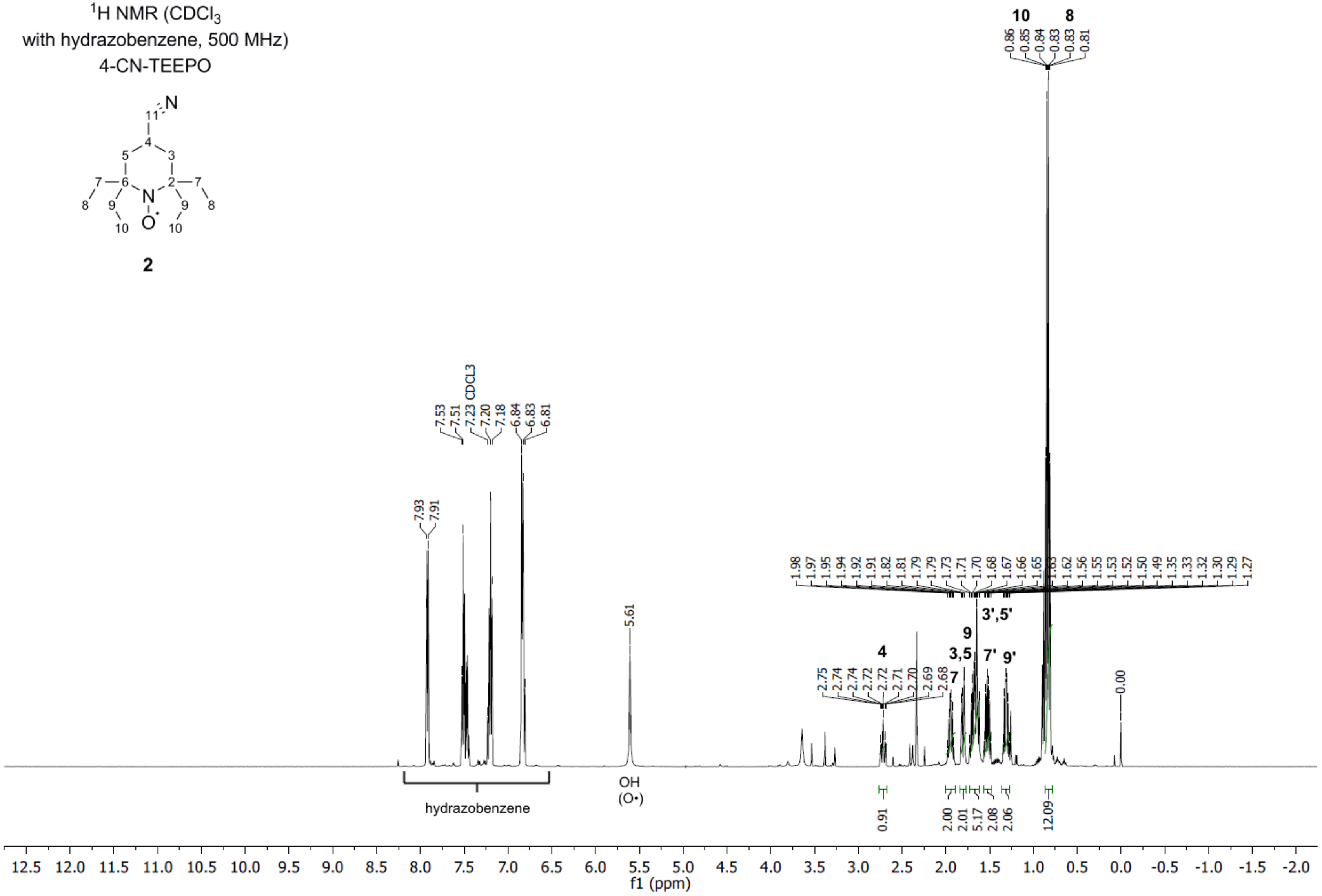

Figure S3. ^{1}H spectrum of 4-Carbonitrile-2,2,6,6-tetraethylpiperidin-1-oxyl (4-CN-TEEPO, **2**) in $CDCl_3$.

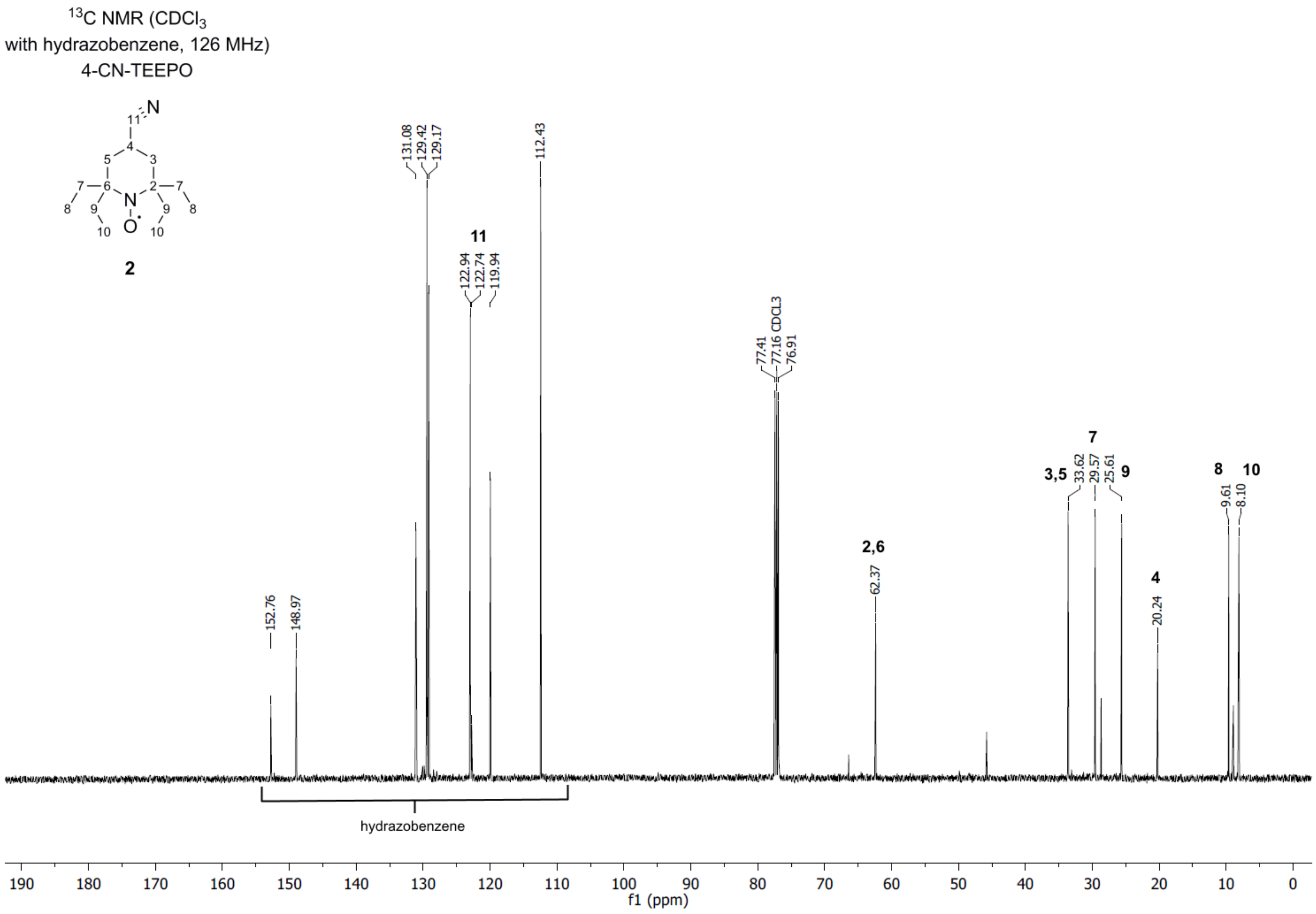

Figure S4. ^{13}C spectrum of 4-Carbonitrile-2,2,6,6-tetraethylpiperidin-1-oxyl ((4-CN-TEEPO, **2**) in $CDCl_3$.

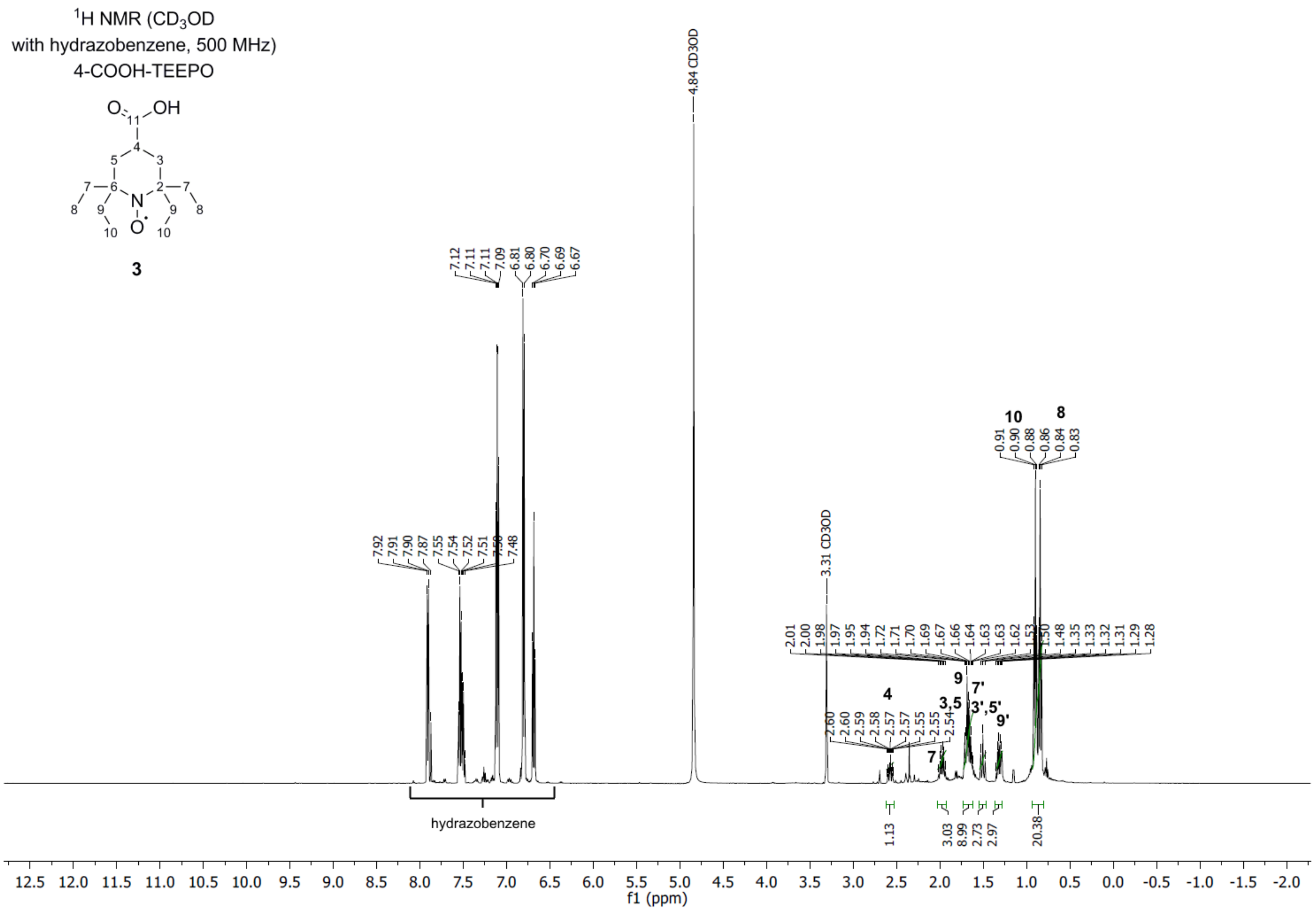

Figure S5. ^{1}H spectrum of 4-Carboxy-2,2,6,6-tetraethylpiperidin-1-oxyl (4-COOH-TEEPO, **3**) in CD_3OD.

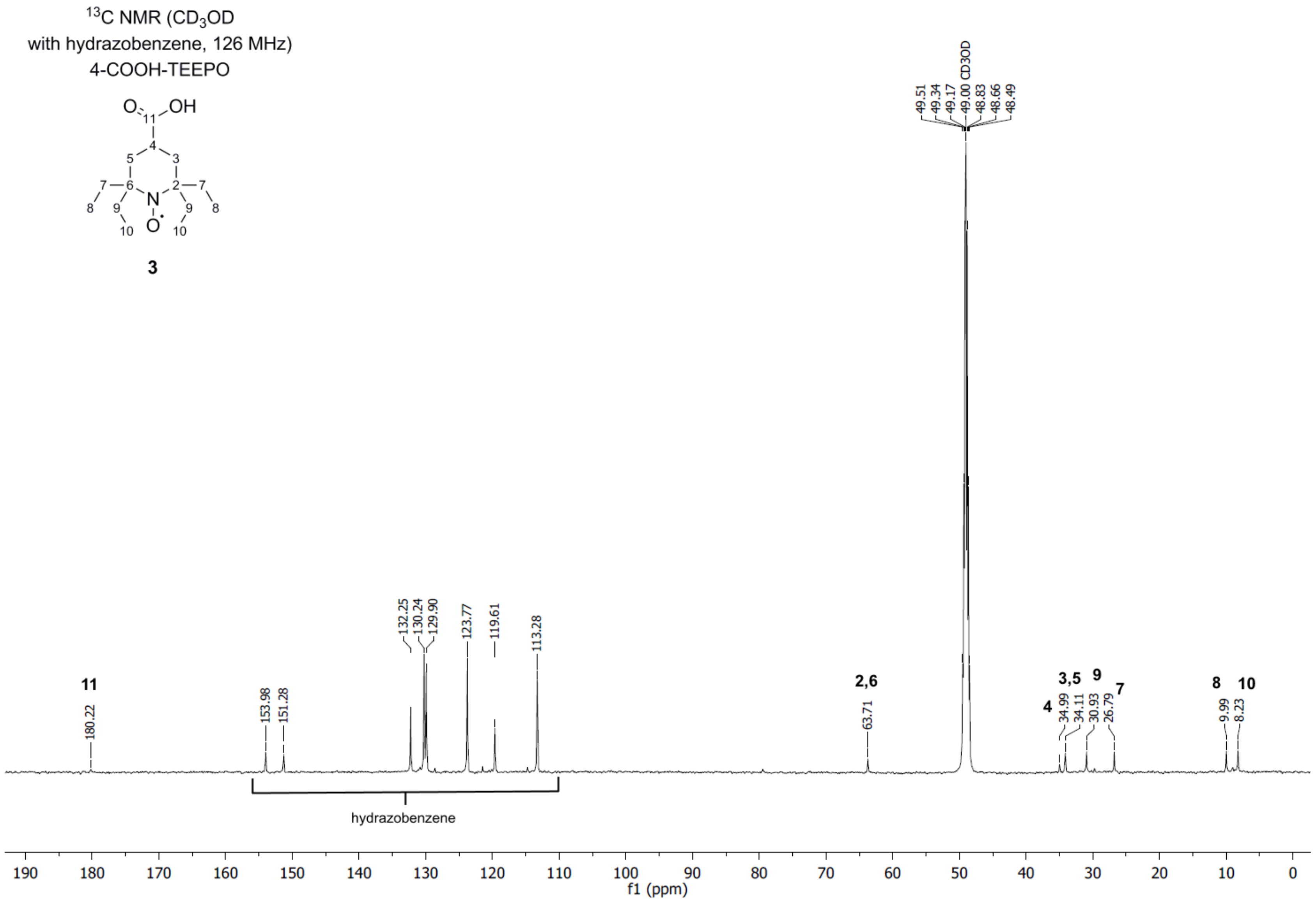

Figure S6. ^{13}C spectrum of 4-Carboxy-2,2,6,6-tetraethylpiperidin-1-oxyl (4-COOH-TEEPO, **3**) in CD_3OD.

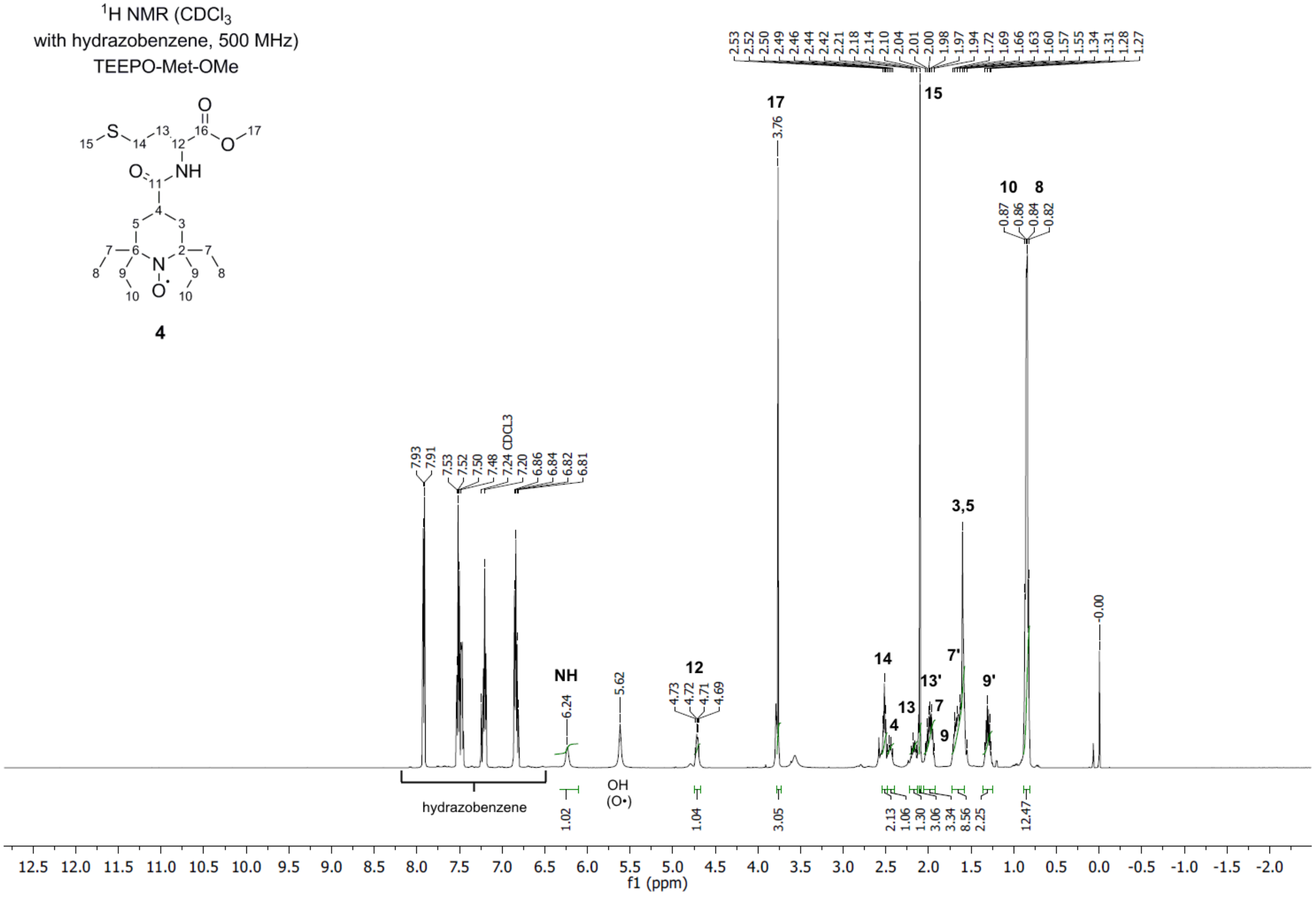

Figure S7. ^{1}H spectrum of 4-[(L-Methionine methyl ester)carbonyl]-2,2,6,6-tetraethylpiperidin-1-oxyl (TEEPO-Met-OMe, **4**) in CDCl$_3$.

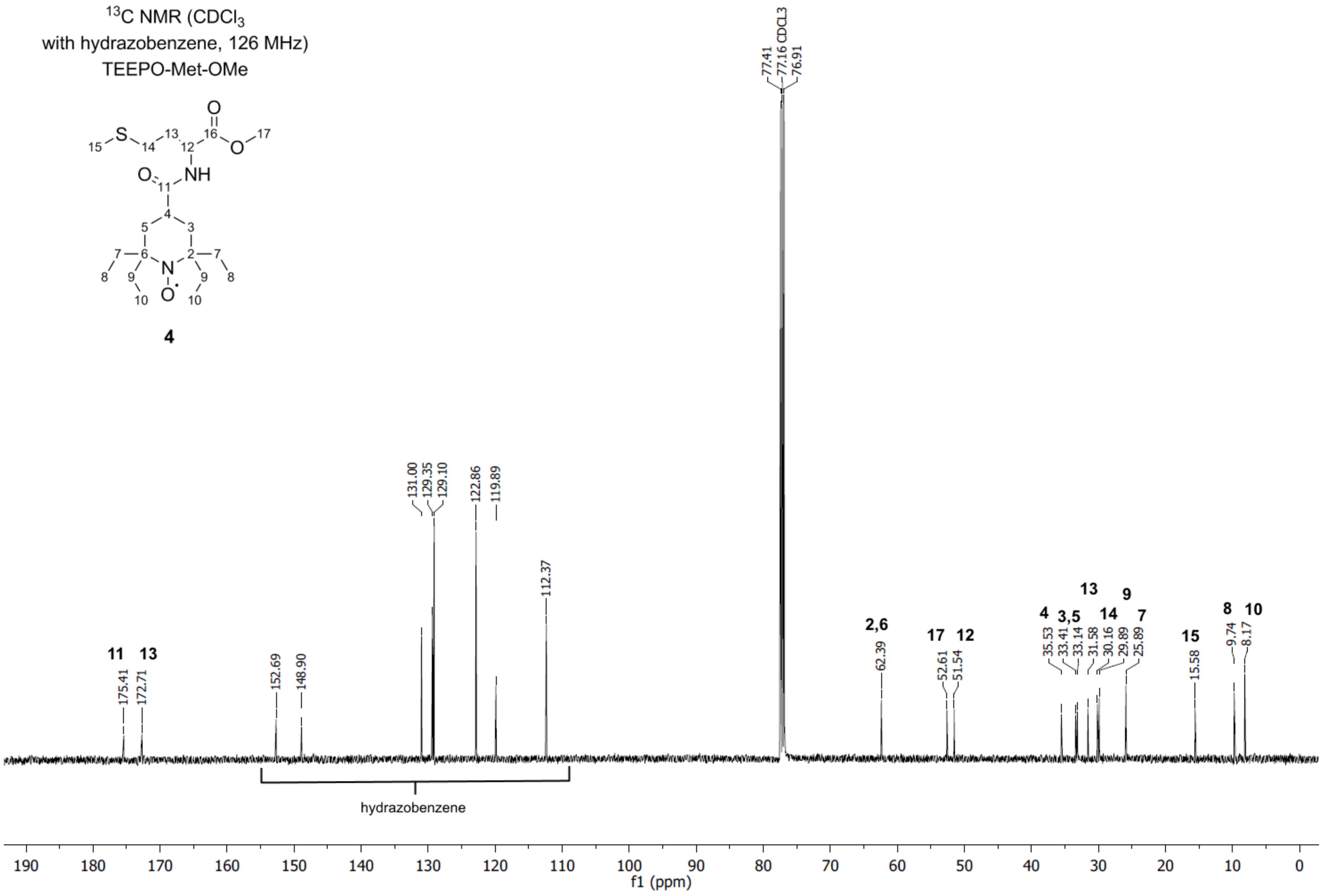

Figure S8. ^{13}C spectrum of 4-[(L-Methionine methyl ester)carbonyl]-2,2,6,6-tetraethylpiperidin-1-oxyl (TEEPO-Met-OMe, **4**) in $CDCl_3$.

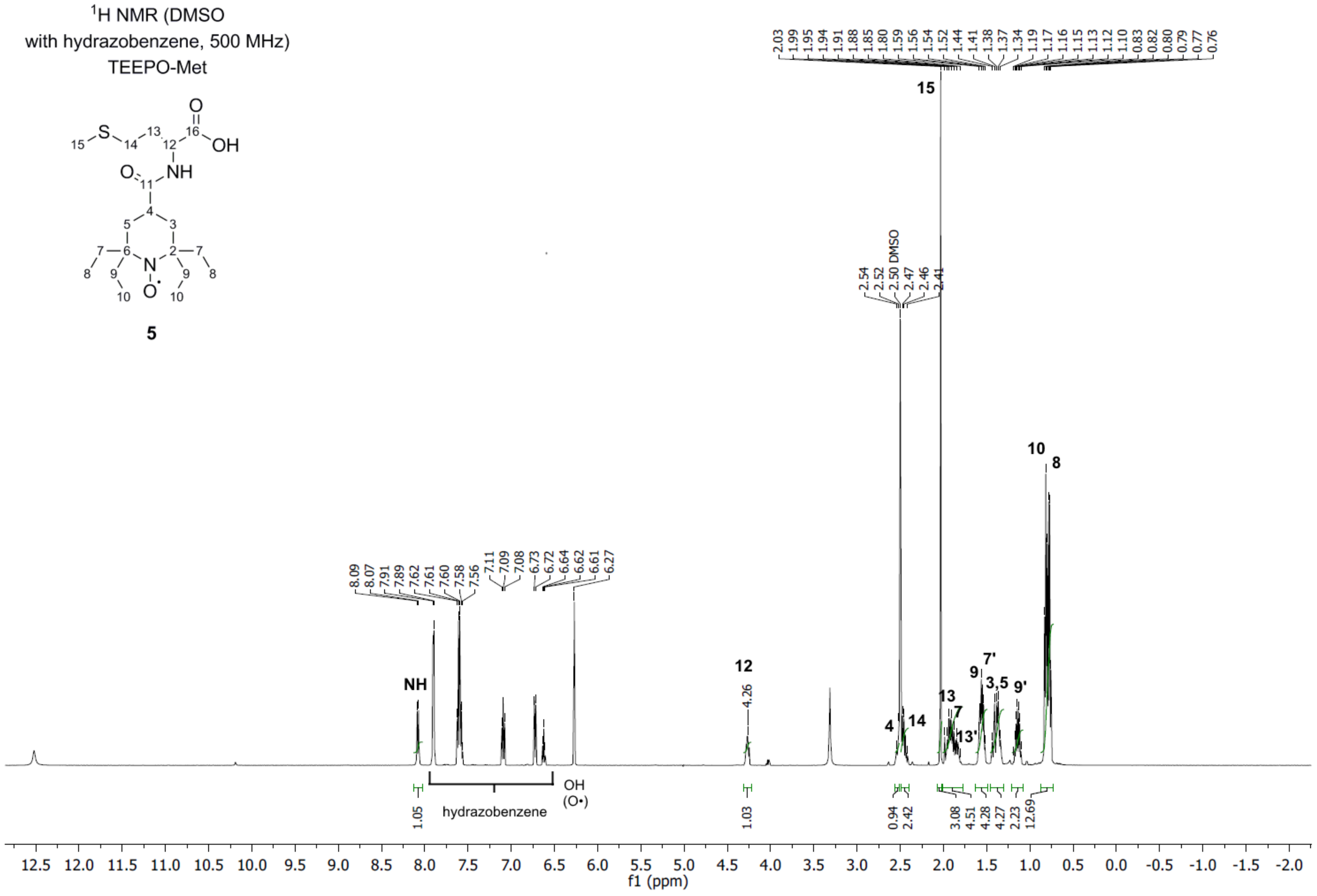

Figure S9. ^{1}H spectrum of 4-[(L-Methionine)carbonyl]-2,2,6,6-tetraethylpiperidin-1-oxyl (TEEPO-Met, **5**) in DMSO.

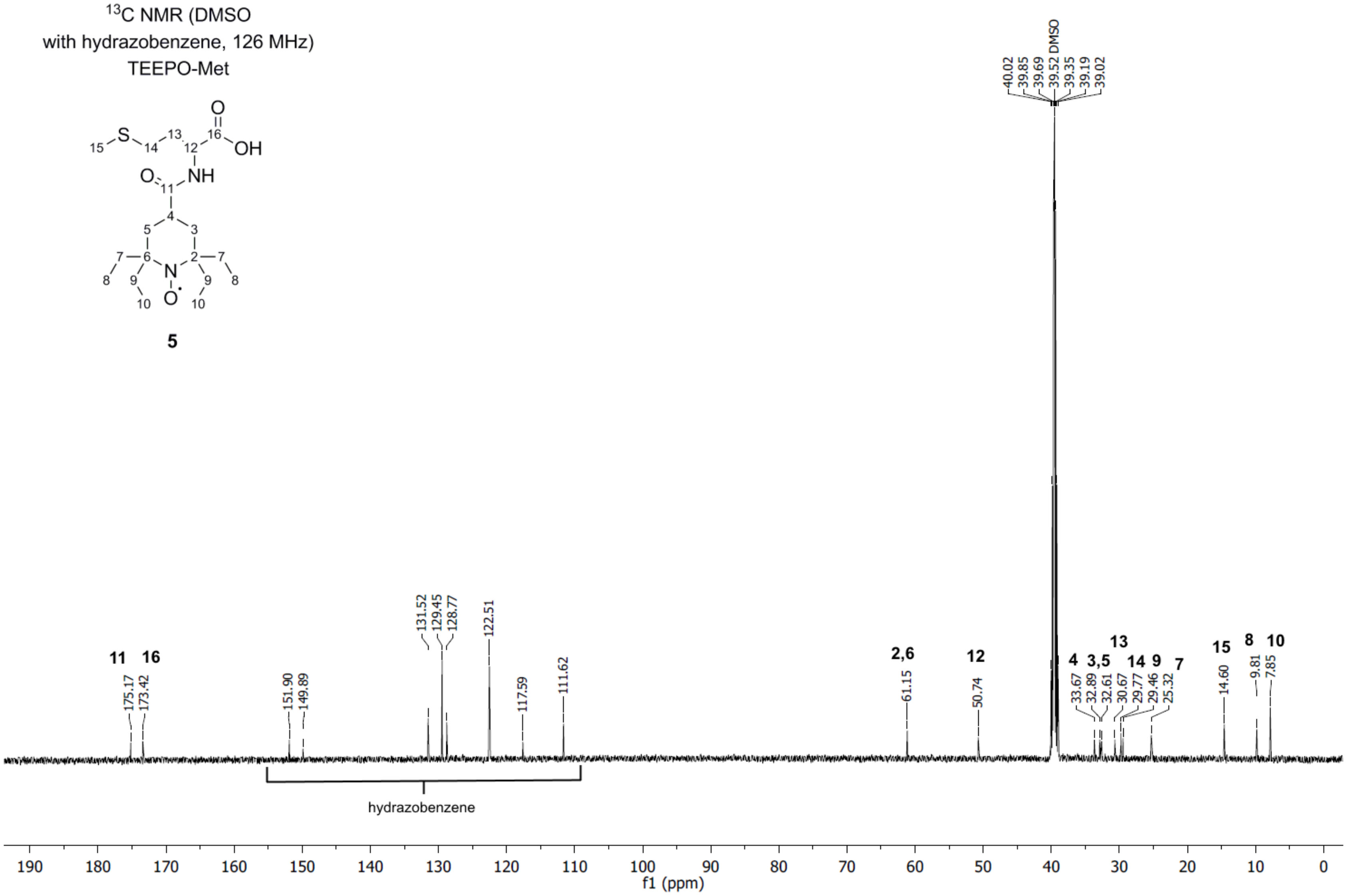

Figure S10. ^{13}C spectrum of 4-[(L-Methionine)carbonyl]-2,2,6,6-tetraethylpiperidin-1-oxyl (TEEPO-Met, **5**) in DMSO.

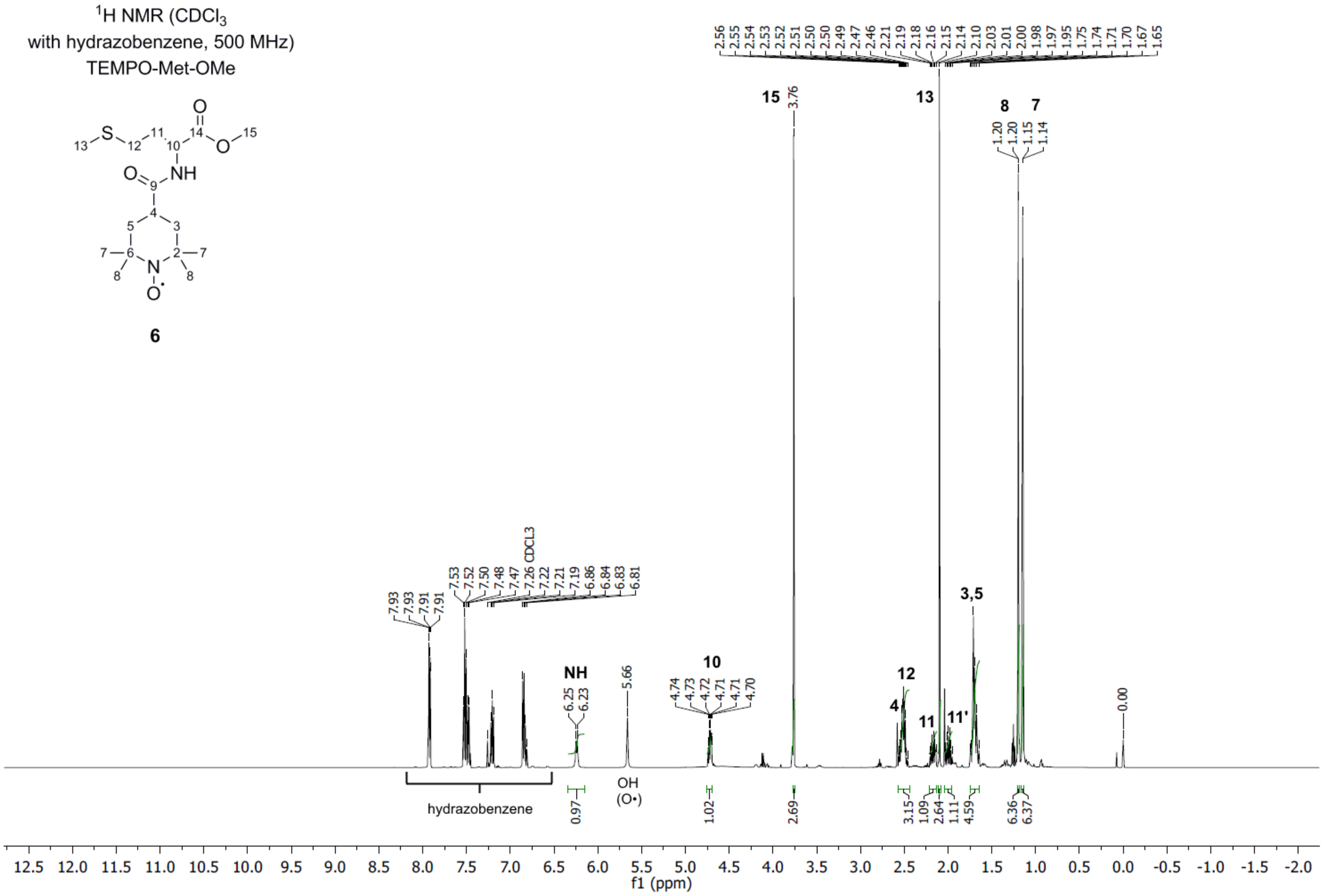

Figure S11. ^{1}H spectrum of 4-[(L-Methionine methyl ester)carbonyl]-2,2,6,6-tetramethylpiperidin-1-oxyl (TEMPO-Met-OMe, **6**) in $CDCl_3$.

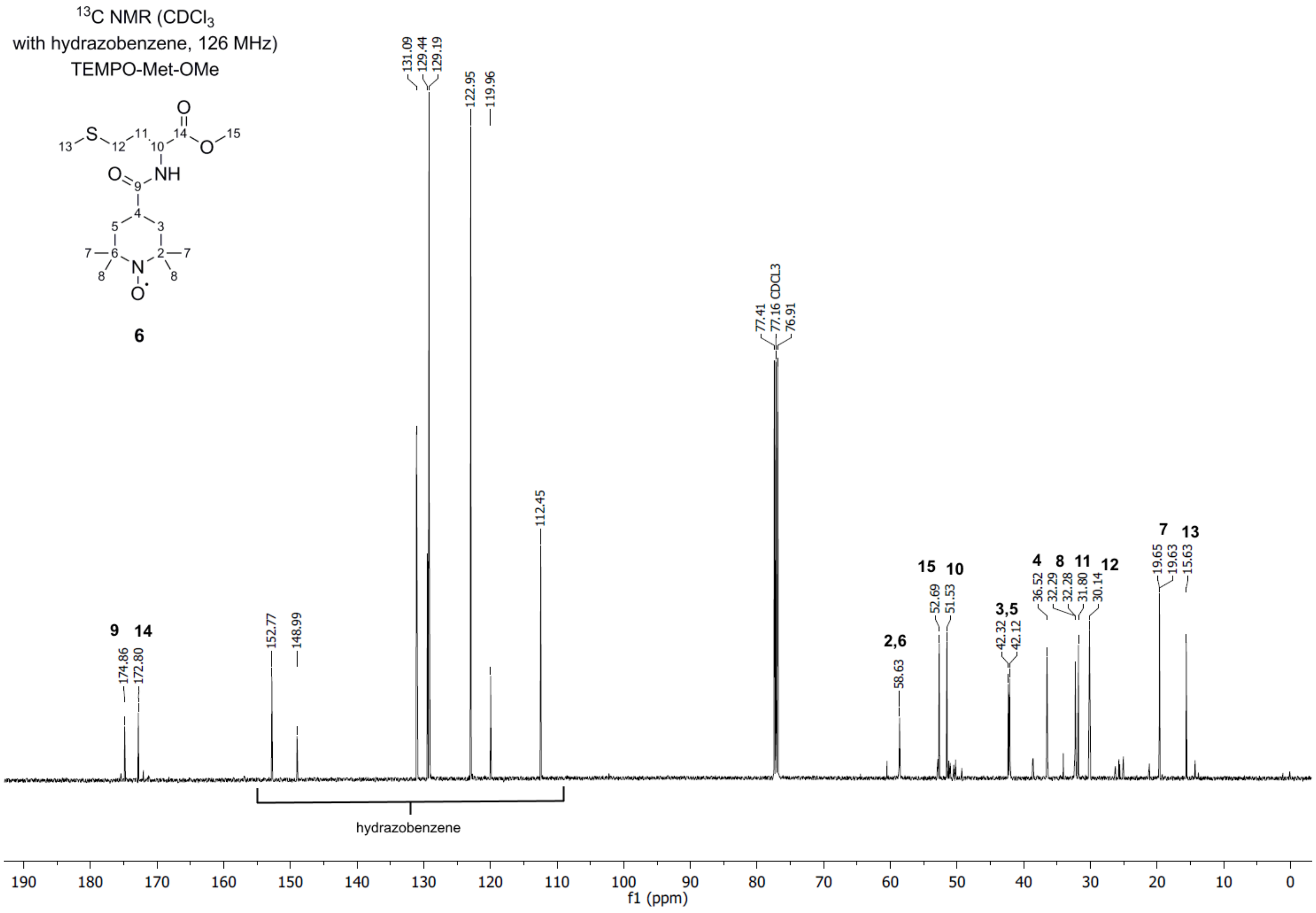

Figure S12. ^{13}C spectrum of 4-[(L-Methionine methyl ester)carbonyl]-2,2,6,6-tetramethylpiperidin-1-oxyl (TEMPO-Met-OMe, **6**) in CDCl$_3$.

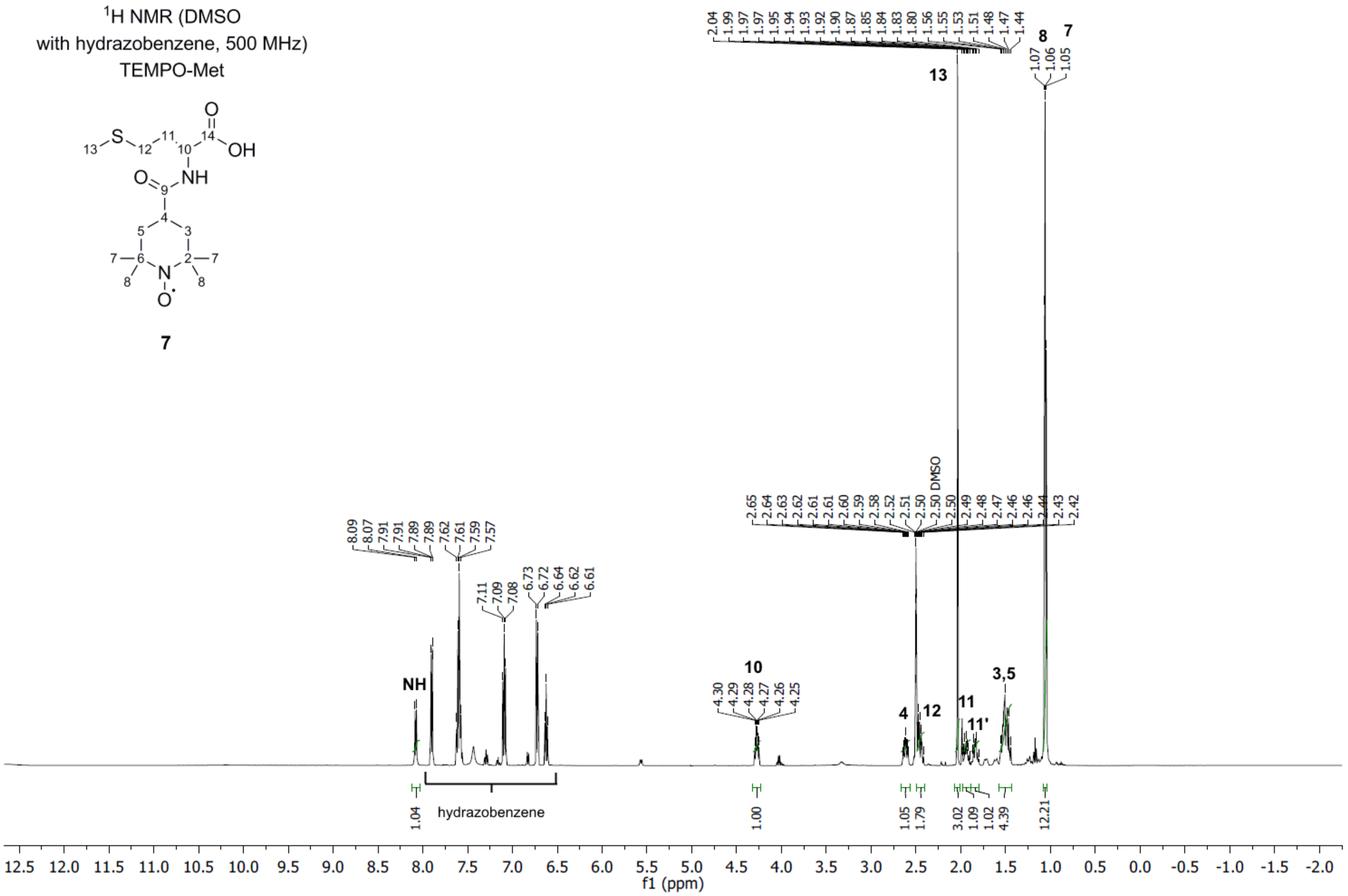

Figure S13. ^{1}H spectrum of 4-[(L-Methionine)carbonyl]-2,2,6,6-tetramethylpiperidin-1-oxyl (TEMPO-Met, **7**) in DMSO.

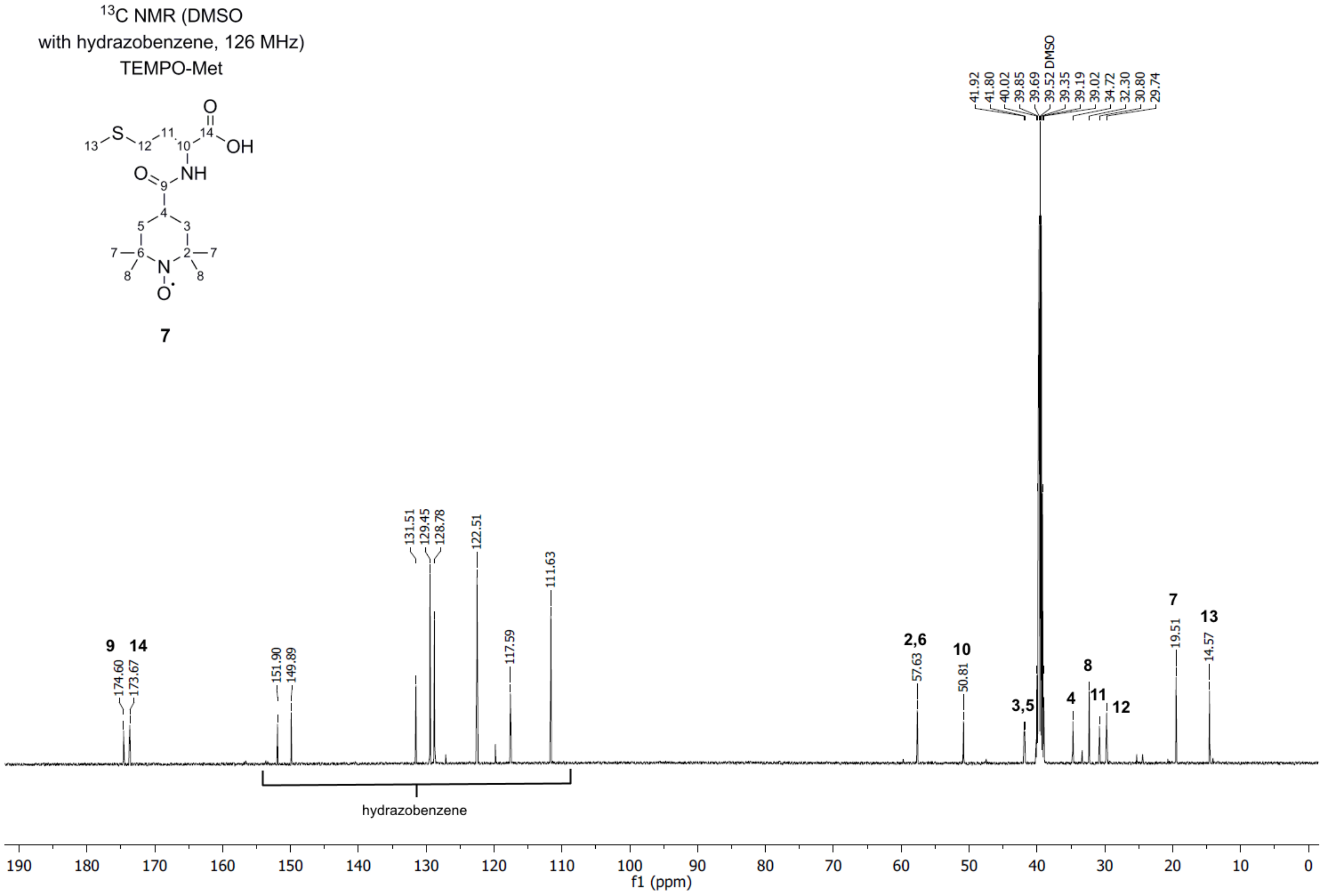

Figure S14. ^{13}C spectrum of 4-[(L-Methionine)carbonyl]-2,2,6,6-tetramethylpiperidin-1-oxyl (TEMPO-Met, **7**) in DMSO.
